# Fertility Preservation Among Patients With Breast Cancer: A Qualitative Study of Patient and Healthcare Provider Perspectives

**DOI:** 10.7759/cureus.89704

**Published:** 2025-08-09

**Authors:** Mounika Polavarapu, Camelia Arsene, Shipra Singh, Jochebed Boakye Ansah

**Affiliations:** 1 Population Health, Pediatrics, and Obstetrics and Gynecology, The University of Toledo, Toledo, USA; 2 Research, Internal Medicine, The University of Toledo, Toledo, USA; 3 Population Health, The University of Toledo, Toledo, USA

**Keywords:** barriers to care, breast cancer patients, fertility preservation, patient referral, qualitative study

## Abstract

Purpose: The aim of this study is to explore barriers impacting the decision about pursuing fertility preservation (FP) among women in the reproductive age group diagnosed with breast cancer before beginning cancer treatment at the patient, healthcare provider, and healthcare system levels from both patients’ and healthcare providers’ perspectives.

Methods: A qualitative study design was performed with a phenomenological approach using semi-structured interviews at a regional cancer institute providing specialty care for breast cancer. The total sample size was thirteen, including eight oncology healthcare providers and five patients with breast cancer.

Results: Prevalent patient-level barriers included (i) financial constraints, (ii) lack of knowledge about FP, and (iii) fear of delay in cancer treatment. Patient-level barriers, as perceived by physicians, also highlighted the financial burden and the overwhelming news of a cancer diagnosis when treatment often takes priority. Common healthcare provider-level barriers were (i) time constraints to independently coordinate a referral process, (ii) limited specialized knowledge about FP, and (iii) assumptions about patients' fertility needs. Finally, the health system-level barriers included a lack of (i) a pre-determined referral pathway for FP and (ii) accessible information about existing resources.

Conclusions: Despite longstanding recognition of barriers to FP among patients with cancer, challenges persist, particularly in regional healthcare settings. Our findings highlight the need for a multi-level approach to enhance FP care for breast cancer survivors, including implementing automated referral systems, enhancing provider education on FP, and addressing financial barriers through targeted policy actions. These strategies could ensure equitable access to FP services and improve patient outcomes.

## Introduction

Globally, the breast cancer burden is expected to rise by 40%, up to >3 million new cases annually by 2040 [1]. Patients with breast cancer are uniquely situated within the broad landscape of cancer survivorship. Advancements in early detection and improved treatment modalities have resulted in longer survival times compared to those with many other types of cancer. This prolonged survival necessitates a heightened focus on post-treatment quality of life, including the possibility of preserving fertility [2]. With the current social trend of deferring motherhood until later in life, an increasing number of women who have not completed childbearing at the time of cancer diagnosis are likely to desire pregnancy following chemotherapy [3].

The field of fertility preservation (FP) has expanded dramatically in the last two decades. It has been attributed to (i) a growing recognition of potential fertility loss as a significant side effect of cancer treatment, and (ii) the development of enabling technologies such as oocyte vitrification and ovarian tissue cryopreservation for subsequent autografting [4]. This has resulted in widespread, albeit uneven, access to FP for many women and young girls. The American Society of Clinical Oncology (ASCO) recommended in its 2018 guidelines and reaffirmed in its 2025 update that FP discussions and referrals should occur before the initiation of cancer therapy whenever feasible [5]. However, substantial gaps between guideline recommendations and real-world practice persist, with recent studies indicating that approximately 50% of patients do not recall discussing FP with them [6]. At the time of diagnosis, about half of reproductive-age women are concerned about becoming infertile or having diminished reproductive function as a result of breast cancer treatment; however, only a tiny minority (10%) pursue FP options [7].

Existing literature has described the barriers toward FP care and attitudes, both at the patient and provider levels [8,9]. More recent studies have also drawn attention to disparities in access to FP services, disproportionately affecting patients based on age, parity, and location of care [10].

While the decision-making process regarding FP has been described among childhood cancer survivors, the synthesis of information lacks specificity for patients with breast cancer. For childhood cancer survivors, the focus is often on addressing the long-term effects of cancer treatment on fertility as a late effect. Unlike childhood cancer survivors who often confront fertility issues upon reaching adulthood [11], breast cancer survivors are typically diagnosed during their reproductive years when fertility is declining naturally for some, amplifying the urgency and relevance of preserving fertility [12].

While general knowledge exists regarding FP in cancer survivors, exploring the specific perspectives and challenges faced by patients with breast cancer is crucial for tailoring appropriate interventions and support systems that address their unique needs. Recent qualitative studies have highlighted the importance of individualized, empathetic fertility discussions that are tailored to patients’ personal values, cultural contexts, and emotional needs following a cancer diagnosis [13].

This study aims to assess the patient-level, healthcare provider-level, and health system-level factors among women diagnosed with breast cancer at a regional healthcare facility, impacting their life-altering decision about pursuing FP before beginning cancer treatment.

Although at the time this study was designed, there was limited literature specifically addressing FP referrals and barriers among patients with breast cancer, subsequent publications, including the study by Carmona et al. [14], have provided important insights. Their primarily quantitative study, based on surgeon and patient reports from a large, pan-Canadian cohort, found that while fertility discussions often occurred, referrals for FP were inconsistently offered, partly due to provider assumptions and system-level barriers such as time constraints and lack of standardized workflows. Building upon these findings, our qualitative study, conducted within a US regional healthcare setting, captures the lived experiences and perspectives of patients with breast cancer and oncology providers, offering a more comprehensive understanding of barriers to FP in settings outside of large comprehensive cancer centers.

## Materials and methods

Study design

We conducted a qualitative study using semi-structured interviews with oncology healthcare providers caring for patients with breast cancer. A phenomenological approach describing "what" and "how" was used to understand the essence of the experiences of study participants with FP discussions and referrals as part of breast cancer treatment plans. The research protocol was approved by the organization's Institutional Review Board (IRB #21-183). Participant interviews were conducted from January to June 2022. Compliance with the COnsolidated criteria for REporting Qualitative research (COREQ) checklist has been presented in the supplemental files.

Setting

The study was conducted at a large regional cancer institute that has served more than 67,000 cancer patients in rural and urban settings over the past 25 years. The institute also provides specialty care for breast, cardio, and lung cancers and offers a survivorship program for post-treatment monitoring and progress.

Eligibility criteria and recruitment

For healthcare providers: Oncology physicians or nurse practitioners who provide care to patients with breast cancer were invited to participate. Purposive sampling was used to balance the provider sample based on their training. The sample size was based on the number of consenting eligible providers at the organization.

For patients: The eligibility criteria for patients were women who (i) were aged 18-49 years, (ii) had been diagnosed with breast cancer in the last five years, and (iii) had either completed or had ongoing cancer treatment. Patients with breast cancer and neurological or cognitive dysfunctions were excluded from the study. All eligible patients served by the cancer institute were invited to participate in the study through phone calls. Interested participants were scheduled to interview via phone, WebEx, or Zoom, as preferred by the patient. After each patient was reached at least once, the final sample size depended on the number of consenting participants. If a phone call was not answered, multiple attempts were made at various combinations of weekdays and times.

Data collection procedures

Interview guides are provided in the online appendix. After obtaining written informed consent, all interviews were recorded and lasted up to 60 minutes. No repeat interviews were conducted. Initially, participants were asked to describe their experiences with the discussion of FP. Furthermore, the interview questions explored four main domains intended to draw out detailed descriptions of patients' and providers' perspectives on the discussion of FP after a breast cancer diagnosis. The four domains were driven by the factors influencing patient-centered communication described by Epstein and colleagues: (i) patient factors, (ii) healthcare provider factors, (iii) health systems factors, and (iv) patient-provider relationship factors [15]. All interviews were audio recorded and transcribed verbatim by the research team.

Data analysis 

A thematic analysis was used to analyze participants' interview transcripts. Information-rich quotes were highlighted and categorized into themes and subthemes separately for provider and patient participants. First, the four authors independently read all the transcripts and open-coded them to develop primary codes in order to capture the entire meaning of the interviews authentically. After completing primary coding for a subset of interviews, differences in coding were discussed in a team meeting, and the process was recommenced.

Four authors collated and analyzed the primary codes to develop secondary codes using the axial coding approach. This allowed for grouping similarly coded data, reducing the number of codes from primary to secondary. Differences in coding were discussed by the team in follow-up meetings, and a consensual list of secondary codes was reached.

All authors together organized the secondary codes into preliminary subthemes. This process included a combination of inductive and deductive approaches, attempting to capture distinct information from individual participants and draw meaningful connections relevant to the research questions. Finally, the subthemes with significant overlap were consolidated into themes. The authors finalized the development of subthemes and themes after three iterations of collective grouping and regrouping of secondary codes. The study aimed to achieve thematic saturation, where no new themes would emerge from additional data collection. However, due to the small sample size, it was not possible to definitively determine if full saturation was reached.

Strategies for ensuring the rigor of findings

Four criteria were used to determine the trustworthiness of our research data: dependability, credibility, confirmability, and transferability. The dependability of data was ensured by fostering consistency across the interviews using a semi-structured interview guide. The validity of the coding system was enhanced by triangulation of researchers, where the four authors independently coded a subset of interviews, and a consensus was established after team discussions. This approach helped establish credibility, the truth value of data. Collectively developing preliminary themes and subthemes enabled the elimination of potential bias related to individual researchers, thereby facilitating confirmability. Finally, transferability was aided by a thorough description of the sample and setting, enabling the readers to assess the applicability of the findings in their context.

Researcher characteristics and reflexivity

Each interview was conducted by one or more of the three female authors (1st, 2nd, and 3rd authors) who are academic researchers (1st author: PhD; 2nd author: MD, PhD; and 3rd author: PhD). All three interviewers have varied levels of prior experience working with patients with cancer and healthcare providers, ranging from 5 to 14 years. The fourth female author (4th author: MPH), who screened and recruited the participants in the study, is a doctoral student in training.

No deliberate attempts were made to establish a relationship with the participants before the study commencement. They had no knowledge about researchers’ personal goals and reasons for doing the research on the topic. However, one of the three interviewers had a prior professional relationship with the healthcare providers; therefore, the interviews were conducted by two interviewers with such participants. The semi-structured interview guide ensured the fidelity of the interviews and limited the interviewers’ bias, assumptions, and interests in the research topic.

## Results

Healthcare providers

Eight healthcare providers were interviewed, including four oncology physicians and four oncology nurse practitioners. They had varied years of experience at the institution, ranging from 2 to 12 years. The sample included four females and four males.

Patients

Out of 268 eligible patients, the research team was able to contact 85, of whom 43 agreed to participate. Before each interview, participants were screened for undergoing permanent contraceptive procedures (e.g., hysterectomy) before the breast cancer diagnosis. Ultimately, only five interviews could be completed, as some participants either did not show up for the interview or failed the screening. Each participant received a $50 gift card after completing the interview. The interviewees' age at the time of breast cancer diagnosis ranged from 31 to 41 years.

Healthcare provider interviews

All themes, sub-themes, and corresponding quotes from healthcare provider interviews are summarized in Table 1.

**Table 1:** Oncology clinician interviews: themes, sub-themes, and quotes

Themes	Quotes
Theme 1: Initial conversation with patients	
Sub-theme 1: Initial patient-provider interaction	“In a female in the childbearing period, uh, yes, it is a must we have to discuss for the preservation.”
	“I guess I don't necessarily have like a fertility preservation conversation with them. I do talk about the risk of infertility with chemotherapy.”
	“Somebody has metastatic breast cancer…. Basically, they have to be on treatment for whatever amount of time they have left. So, kind of…fertility preservation does not take priority in these cases.”
	“I identify who are the patients that would be candidate to have a discussion, clearly not for post menopause and also for Pre-menopause, …. you will find out if they are interested ….and 40 years old and so I stop right there.”
	“…… I’ve come across women who have had any real interest in fertility preservation in some ways. Instead, they’re happy to see their fertility go away…honestly, no women have been interested at all. So, I haven’t developed a specific strategy for that.”
Sub-theme 2: Individualized referral process	“Good to maybe have a format and then we can follow in future instead of, you know, jumping on it randomly.”
	“I had a metastatic patient that was obviously motivated to avoid pregnancy... that wasn't a concern of theirs at all.”
Sub-theme 3: Educational/informational materials for patients	“People take these brochures and read; sometimes they don't understand what it means.”
Theme 2: Barriers	
Sub-theme 1: Provider level barrier	“Sometimes comfort level and talking about……not necessarily pregnancy, but sexuality.”
	“Sometimes I refer her, but they think at the fertility clinic, think, gosh is just a regular patient, “So, we're going to give an appointment in 3 weeks or 4 weeks”
	“If we have a formal path and these are triggers patient in need of fertility consultation, and we just follow that path and, uh…. send out that alert and we initiate it automatically.”
Sub-theme 2: Organizational barriers	“Which patient they're going to talk about fertility preservations, those are younger patients and of childbearing age and that have early-stage cancer.”
	“Maybe that extra level of care saying, hey, we understand that you're already facing this really difficult challenge.”
Sub-theme 3: Patient level barriers perceived by physicians	“Okay, I would rather deal with cancer, the treatment right now, and I don't care for the future, and I do not have money for fertility preservation.”
	“They've gotten everything's thrown at them, so they may not even think about it (FP) …. we try to go through that with them, but you don't touch on it in depth. Because there's a lot of information and other things going on at the same time.”
	“If there is a scenario where they're going to be risking their health outcomes for the possibility of some kind of maybe...unknown process to pursue possibly an effective....you know, fertility preservation, that it's not worth the hassle.”
	“They're, they're probably thinking my life, uh, which is maybe more of a tangible thing, uh, versus an uncertainty of, of something that could maybe happen down the line.”
Theme 3: Benchmark quality measure for care related to FP	
	“Generally, that quality question or ways to check for quality, they have their limitations. Yes or no does not reflect the quality of the discussion and the effort that was put into it.”
Theme 4: Future recommendations	
	“I think there should be something that comes up to for sure, such as an alert for women of childbearing age. So, I'm wondering if, like, you're putting in a treatment plan, and something like that would automatically alert you.”
	“It would be nice to have it built right into the treatment plans that, you know, need to address for fertility.”
	“If we have a designated staff with more experience and know how to provide the information and how to triage and just like our yeah…. genetic counselor they know how urgencies genetic tests should be doing right now, because it may affect the surgical options. If we have a similar type of person for fertility, that'd be very helpful. I would ...I would love to have a person like that to give us information right at that time to us and also to patient.”

Theme 1: Initial Conversation With Patients

Sub-theme 1 - initial patient-provider interaction: Significant variability was reported during the initial patient-provider interaction. One provider agreed it is essential to talk about FP, especially with women of childbearing age. Still, most providers reported addressing it indirectly as infertility while discussing treatment options and chemotherapy. Most providers concurred that discussing FP was dependent on the patient's age, parity, and urgency of starting the treatment. Also, there were distinct differences in communication between patients with metastatic and early-stage cancer. Interestingly, two out of eight providers reported that they believed that most patients were not interested in FP, as the patients’ main concerns were in regard to cancer treatment. The nurse practitioners said that their initial interaction with the patients is after the start of treatment, when FP is not the priority. Patients meet with the physician at the initial visit after the breast cancer diagnosis. However, they review medical records and order referrals to the reproductive endocrinologist if needed.

Sub-theme 2 - individualized referral process/differences in patient motivation: Inconsistency in the referral processes among providers was also reported. Three providers reported no knowledge of a referral system in their healthcare system for FP and no specific strategy for referring clients to reproductive medicine clinics. Few providers reported that they believed the patients were not interested in the referral process and were motivated to avoid pregnancies, especially if they had received an unfavorable prognosis. 

Sub-theme 3 - educational/informational materials for patients: Only one provider reported that their institution provides patients with educational material on FP. However, they preferred interacting with their patients directly rather than handing out brochures. In contrast, five providers reported that the healthcare institution provided no standardized educational materials on FP to their patients. The only educational materials readily available were centered on infertility as a side effect due to cancer treatment. 

Theme 2: Barriers

These are grouped into three levels: provider, healthcare system, and patient level as perceived by providers.

Sub-theme 1 - provider-level barriers:** **Some providers expressed their concern regarding the time taken for the FP referral to be processed and completed, which can delay the start of cancer treatment. One provider shared that a lack of specialized knowledge about the FP process is a barrier to discussing FP. Most providers concurred that the financial burden of cancer treatment was a major reason for their hesitancy to initiate discussion about FP, as it was an added cost to patients. The discomfort with discussing sexuality was also identified as a barrier to initiating FP discussion by a provider.

Sub-theme 2 - healthcare system barriers: Most providers were unaware of any comprehensive institutional resources, services, or information regarding FP. One provider reported that some educational brochures were shared with younger patients of childbearing age with early-stage cancer. Additionally, two providers expressed concerns about the lack of institutional-level support for patients regarding their mental health, as they processed and maneuvered all the challenges of different referrals during their treatment. 

Sub-theme 3 - patient-level barriers as perceived by providers: Financial resources were identified as a key barrier for patients to pursue FP by most providers. They expressed concern with initiating FP discussions with patients experiencing high stress and fear in addition to overwhelming information regarding cancer diagnosis and treatment steps. Four providers reported that patients were more interested in prioritizing cancer treatment and getting healthy than FP.

Theme 3: Healthcare System Benchmark Quality Measure for Care Related to FP

More than half of the providers had no knowledge about the current ASCO benchmark quality measure at their healthcare system regarding FP. All providers agreed that it is an essential measure at the healthcare system level to document "yes" or "no" for each patient if FP was discussed with them. However, one provider identified the limitations of these system-level measures as they do not capture the quality of the discussion between a patient and their provider. 

Theme 4: Future Recommendations 

Most providers recommended establishing a standard referral process at their institution that streamlines and automates the FP referral process, reducing the burden on providers. Another important recommendation was for providers' education, as they identified that more education on the FP and the referral process is needed. The designation of support staff with experience in connecting patients with information and triaging all the referral processes was also emphasized.

Patient interviews

All themes, sub-themes, and corresponding quotes from patient interviews are summarized in Table 2.

**Table 2:** Interviews with patients with breast cancer: themes and quotes

Themes	Quotes
Theme 1: Initial conversation with providers- Patient- provider interaction regarding FP	“First oncology doctor and my surgical oncologist…they kind of put their heads together…they recommended the best course of treatment in their eyes with my fertility preservation.”
	“I'm one who likes to make an informed decision and have all the options presented to me and it, it just was not discussed.”
	“I would say that it was just rushed, and people just assumed that I wanted kids; like there was no question that was asked.”
	“I asked, you know if I would be able to have children and basically, he said, you know, since I was pregnant, there wasn't any way to obviously preserve fertility because I was currently pregnant and that that would be something we further explore later.”
Theme 2: Patient satisfaction with providers and the healthcare system	“I very much researched on my own and I actually got a 2nd opinion, so I felt like I got as much information as possible for me to make an informed decision of how I wanted my care to look like.”
	“Very discouraging and not supportive in nature. And you have a lifelong relationship with your oncology team, and I felt like my needs couldn't be met here. So, I called in a couple favors, got a referral to X and ended up just loving my journey there and had a great success rate because of that.”
	“I think it was a lack of communication, to be honest with you. It just didn't. It just didn't feel good.”
	“Like no one asked me, do you want to preserve eggs? It's no one asked like, what do you think about this like, you know kind of like, you know making that informed decision you have to have all of that like risk and benefit and like understand all of your different pathways of option.”
	“And it seemed like they had our best interest in mind. So that made it a little bit of an easier decision.”
Theme 3: Reasons for not Pursuing FP	“I had this care conference with all of the doctors, but I didn't know what my diagnosis was yet before that happened. So, like they immediately, like, they just walked in and started talking about chemotherapy. And I was like, what are you talking about? Like, I didn't know…Like there was missing pieces, so it was kind of like constant shock factor because I didn't have the communication before.”
	“Yeah, I just didn't want to put my body through anymore and I had such rough miscarriages prior to the cancer. I just felt my body had been through enough and I didn't want to take any more risk.”
	“I was already about 41 you know, and I already had 3 children. I was at a different place, not that I wouldn't have been open to having more kids.”
	“I'm educated, and I have two master’s degrees. I've worked in healthcare for my entire career. I know the systems and I still struggled with all that. I can't imagine being of a lower socioeconomic status, maybe having learning difficulties, poor support, and poor financial means.”

*Theme 1 *: *Initial Conversation With Providers (Patient-Provider Interaction) *

Two of the five patients interviewed reported discussing FP with their physicians but shared different experiences. Both patients said that the physician initiated the conversation; however, one patient reported feeling rushed and felt that the provider had preconceived assumptions regarding her reproductive choices. The other patient had a meaningful FP discussion with the physician and the healthcare team. Of the other three patients who did not have the discussion, two reported not having the discussion at all. The last patient was pregnant at the time of diagnosis and decided to initiate the FP conversation as an option later. 

Theme 2: Patient Satisfaction With Providers and the Healthcare System 

Most patients did not have the expected experience with their providers and the healthcare system regarding FP. Lack of communication regarding FP options, risks and benefits of the procedure, and time to process the diagnosis were their significant concerns. One patient researched information on FP on her own and sought a second clinical opinion at a different organization to make an informed decision. Contrary to the other four, one patient shared a positive experience with her provider, who offered appropriate information and resources that existed within the healthcare system. 

Theme 3: Reason for Not Pursuing FP 

All the patients unanimously reported that cost was a significant reason for potentially not pursuing FP, as it was not covered by insurance. Three patients expressed that the urgency of starting treatment by the healthcare team took precedence over exploring information about FP. Patients also identified fear of stressing the body and the emotional impact of the cancer diagnosis that prevented them from pursuing FP. Two patients shared that having kids previously and being older in age were instrumental in their decisions. More than half of the patients recognized the impact of socioeconomic factors such as education level and employment status as significant barriers to FP. Having higher education and working in the healthcare system supported one participant in advocating for her fertility decisions.

## Discussion

This study offers a unique, multi-level examination of barriers to FP among patients with breast cancer within a regional healthcare setting, where FP practices have been less systematically characterized in the literature than large academic or comprehensive cancer centers [10]. The findings can be contextualized through the lens of the competing demands model, which illustrates how both patients and healthcare providers must navigate multiple, often conflicting priorities [16].

The findings of our study reaffirm longstanding barriers to FP identified in prior literature [6,8,17-19] but importantly demonstrate how these challenges continue to persist across patient, provider, and healthcare system levels in regional facilities. For patients, the urgency of initiating cancer treatment frequently conflicted with the desire to preserve fertility, while providers often balance time constraints, the immediacy of cancer treatment, and limited knowledge about FP. System-level challenges, such as the absence of standardized referral pathways, educational materials, and comprehensive institutional support, further compounded these barriers.

The interconnectedness of barriers across patient, provider, and healthcare system levels highlights the complexity of effectively addressing FP in breast cancer care. Isolated efforts targeting one level are unlikely to yield significant improvements without parallel changes across all levels. For example, at the patient level, financial constraints, lack of awareness, and emotional distress may deter individuals from actively seeking FP options [20]. These barriers are amplified by provider-related factors such as inadequate knowledge about FP, discomfort in discussing reproductive health, and lack of time to communicate FP options effectively [21,22]. Further, systemic issues such as the lack of standardized referral pathways, limited educational materials, insufficient insurance coverage for FP, and inadequate mental health support contribute to the overall barriers to FP [17,23]. Therefore, it becomes apparent that addressing one level of barrier alone is insufficient, as they are interconnected and mutually reinforcing. A comprehensive approach that simultaneously targets patient, provider, and system-level factors, including improving institutional resources, providing targeted provider education, and implementing financial support mechanisms, is crucial for promoting and facilitating FP among patients with breast cancer.

Standardized referral pathways for FP have been recommended in the literature, particularly involving multidisciplinary care teams for pediatric patients with cancer [24]. ASCO recommends that patients with cancer at risk of infertility consult a fertility specialist before starting cancer treatment to understand all the available options [25]. Although many National Cancer Institute-designated comprehensive cancer centers have adopted and implemented interventions to address FP, gaps in consistency and access remain [26]. Additionally, it is important to acknowledge that many patients lack access to such specialized institutions. Our findings indicate that challenges persist in regional healthcare facilities, similar to the setting of our study. Efforts should be made to disseminate best practices from ASCO guidelines and comprehensive care centers to regional facilities, ensuring that patients with breast cancer receive consistent and high-quality information and support regarding FP.

As documented in the literature [27,28], the cost of FP has been unambiguously acknowledged by both patients and providers as a significant barrier in our study. This barrier has policy implications beyond a single healthcare system. The cost barrier could be alleviated through policy action mandating the coverage of FP and infertility treatments by both private and public insurance. Only 17 US states have passed laws that require insurers to either cover or offer coverage for infertility diagnosis and treatment. New York passed a bill in 2019 requiring in vitro fertilization (IVF) and FP services for comprehensive private health insurance policies [29]. It has been estimated that the increase in premiums would be 0.5%-1.1% and 0.02% for mandating IVF coverage and FP, respectively [29]. Research has shown that states with laws mandating insurance coverage of FP have significantly higher rates of fertility counselling before chemotherapy [30].

In addition to policy change, greater emphasis should be placed on sharing existing support resources with patients. Healthcare systems can improve the sharing of existing resources for patients with cancer, such as the Livestrong Fertility Program, which offers information, resources, and financial support for cancer survivors whose fertility has been impacted by cancer or its treatment. Similarly, the Alliance for FP provides a list of financial assistance programs in the form of grants and financing to help patients with cancer offset some of the costs of FP. Nurse navigators already engaged with patients and caregivers at the institution could help disseminate information on these available resources.

More broadly, addressing FP barriers requires a patient-centric approach that integrates education, emotional support, and empowerment. Tailored education programs can help patients make informed decisions about FP and understand available financial assistance programs. Concurrently, the emotional toll of a cancer diagnosis, further exacerbated by potential infertility, requires emotional and mental health support toward FP services. Discussing resources such as the Livestrong Fertility Program and the Alliance for FP during initial FP conversations may help reduce information gaps. Finally, promoting patient advocacy through education and empowerment initiatives could encourage patients to take a proactive role in FP discussions, thereby strengthening patient-provider communication. Collectively, such a comprehensive patient-centric approach could help address the interconnected barriers to FP.

Strengths and limitations

The limited sample size is one of the primary limitations of this study. Additionally, mixed interview modes (Zoom and telephone) may have introduced variability in participants’ responses. However, these modalities were necessary to broaden our reach and accommodate patients’ convenience during the COVID-19 pandemic (at the time of data collection). The rigor and trustworthiness of our results were ensured by dependability, credibility, confirmability, and transferability. Another limitation of the study is recall bias. A new breast cancer diagnosis is often paired with a combination of emotional responses that may bias the recall of events during the interviews. A strength of this study is that we conducted interviews with patients and healthcare providers from the same setting in order to understand barriers to FP among patients with breast cancer at length. This comprehensive understanding included personal perspectives and practical challenges at the levels of the patient, clinician, and healthcare system. Information and insights provided by our study could benefit similar settings in developing a multi-level approach to improving oncofertility care among patients with breast cancer and survivors.

## Conclusions

In conclusion, our study results have demonstrated that addressing FP barriers necessitates a holistic approach, appreciating the interplay of patient, provider, and system-level challenges. Such targeted interventions and system-wide changes may ensure equitable access to FP services for all patients with breast cancer. While there is substantial literature on FP among patients with cancer, our study highlights gaps that still exist in specific areas, such as how emerging trends in patient preferences, advancements in FP technologies, and disparities in access based on geography or socioeconomic status might affect decision-making. Although our study did not explore these aspects directly, it provides a timely examination of the barriers faced by patients and healthcare providers in the current healthcare landscape.

## Appendices

Questions for oncology clinicians*

1. Do you discuss “fertility preservation” with your newly diagnosed breast cancer patients?

2. Could you walk us through the process you follow while discussing fertility issues with a newly diagnosed breast cancer patient?

How do you present the discussion about fertility preservation to your newly diagnosed breast cancer patient? Asking the patient whether they want to become pregnant in the future, or brief overview (2 sentences), or pamphlet, or leaving it up to the reproductive endocrinologist.

3. If referred, do you still discuss fertility preservation with your patients? The significance of the referral, how they will help the patients.

4. What are the most common challenges you face as a clinician caring for breast cancer patients (oncologist or nurse practitioner) when approaching the subject of fertility preservation?

5. What are the most common challenges that you believe your patients face in making a decision regarding fertility preservation? (e.g., high risk of cancer recurrence, risk to the infant, and delay in treatment)

6. Frequency of asking about fertility preservation during the process of cancer treatment? Only at the beginning or continue during follow-ups after treatment, when a patient comes for the next 5 years? 

7. Are there any resources available at your organization to support newly diagnosed breast cancer patients making a decision about fertility preservation? What are they?

8. In your experience, what additional support do patients seek in making a decision regarding fertility preservation?

a. Family decision (culture, religion, financial)

9. In your opinion, what additional resources should be provided for patients toward fertility preservation?

10. In your opinion, what challenges remain in patients receiving information and discussion about their fertility risks and options to fertility preservation prior to beginning the treatment?

11. What are your thoughts about QOPI quality measure of “Did you discuss fertility preservation with the patient: Yes/No”?

12. Is there something else you would like to add on this topic? (Unique experiences with patients, unusual circumstances about fertility preservation)

*May include more probing questions based on the participant responses.

Questions for patients*

1. What were your plans about getting pregnant before you ever received your cancer diagnosis?

2. What were your plans for getting pregnant after you received your cancer diagnosis?

3. If your plans changed, what were the reasons? 

4. Do you have an understanding of the term “fertility preservation”?

5. Did your oncologist discuss fertility preservation with you prior to starting the cancer treatment? (Skip to question 7, if answered no) 

6. What information did your oncologist provide you regarding fertility preservation?

7. Were you referred to any other provider for the discussion on the topic of “fertility preservation”?

If yes:

a. Who were you referred to? Are they within Promedica or outside?

b. Could you describe your experience talking to the expert? Did you receive all the information necessary to make the decision? What information did you receive?

8. Did your oncologist assist you in making a decision about fertility preservation? How?

9. What information was helpful to you in making a decision? And what information was not helpful to you? 

10. Were any alternative options discussed with you pertaining to fertility preservation? What alternatives were they?

11. Did you have any concerns about undergoing fertility preservation?

12. What concerns did you have during the decision-making process (if any)? Were they alleviated/not alleviated? How were they alleviated?

13. Were any resources offered to you at the hospital to assist you with making a decision? 

14. What were they? 

15. What additional resources would you have liked to have?

16. Did this decision-making process include anyone besides you, such as family, friend, partner, etc.? How much did these relationships influence your decision about “fertility preservation?”

17. What was the role of your cultural background (religion, race/ethnicity) in the decision-making process regarding fertility preservation? How does it influence your decision about “fertility preservation”?

18. Did you experience any barriers to pursuing fertility preservation? 

19. What were they?

20. Did your health insurance cover fertility preservation?

21. Is there something you would like to add on this topic that we may not have covered?

*May include more probing questions based on the participant responses.

Supplementary file

COREQ checklist: A checklist of items that should be included in reports of qualitative research. 

**Table 3:** COREQ checklist COREQ, COnsolidated criteria for REporting Qualitative research

Topic	Item No.	Guide Questions/Description	Reported on Page No.
Domain 1: Research team and reﬂexivity			
Personal characteristics			
Interviewer/facilitator	1	Which author/s conducted the interview or focus group?	5
Credentials	2	What were the researcher’s credentials? E.g., PhD and MD	5
Occupation	3	What was their occupation at the time of the study?	5
Gender	4	Was the researcher male or female?	5
Experience and training	5	What experience or training did the researcher have?	5
Relationship with participants			
Relationship established	6	Was a relationship established prior to study commencement?	6
Participant knowledge of the interviewer	7	What did the participants know about the researcher? E.g., personal goals and reasons for doing the research	
			6
			Interviewer characteristics	8	What characteristics were reported about the interviewer/facilitator? E.g., bias, assumptions, and reasons and interests in the research topic	
6					
Domain 2: Study design					
Theoretical framework			
Methodological orientation and theory	9	What methodological orientation was stated to underpin the study? E.g. grounded theory, discourse analysis, ethnography, phenomenology, and content analysis	
			4
			Participant selection			
Sampling	10	How were participants selected? E.g., purposive, convenience, consecutive, and snowball	
			4
			Method of approach	11	How were participants approached? E.g., face-to-face, telephone, mail, and email	
4					
Sample size	12	How many participants were in the study?				6
Non-participation	13	How many people refused to participate or dropped out? Reasons?	6
Setting			
Setting of data collection	14	Where was the data collected? E.g., home, clinic, and workplace	4-5
Presence of non-participants	15	Was anyone else present besides the participants and researchers?	
			4-5
			Description of sample	16	What are the important characteristics of the sample? E.g., demographic data and date	
6					
Data collection					
Interview guide	17	Were questions, prompts, and guides provided by the authors? Was it pilot tested?	4
			Repeat interviews	18	Were repeat interviews carried out? If yes, how many?	4
Audio/visual recording	19	Did the research use audio or visual recording to collect the data?	4-5
Field notes	20	Were ﬁeld notes made during and/or after the interview or focus group?	4-5
Duration	21	What was the duration of the interviews or focus group?	4
Data saturation	22	Was data saturation discussed?	5
Transcripts returned	23	Were transcripts returned to participants for comment?	No
